# Langerhans Cell Sarcoma: A Case Report Demonstrating Morphological and Immunophenotypical Variability within a Single Lesion

**DOI:** 10.1155/2017/9842605

**Published:** 2017-05-24

**Authors:** Rasika Singh, Charles Edward Keen, Christopher Stone, Patrick Sarsfield

**Affiliations:** ^1^Department of Cellular Pathology, Plymouth Hospitals NHS Trust, Plymouth PL6 8DH, UK; ^2^Department of Cellular Pathology, Royal Devon and Exeter NHS Foundation Trust, Old Pathology Building, Church Lane, Exeter EX2 5DW, UK; ^3^Department of Plastic Surgery, Royal Devon and Exeter NHS Foundation Trust, Exeter EX2 5DW, UK

## Abstract

Langerhans cells are antigen presenting dendritic cells and tumours arising from these are rare. The tumours arising from these dendritic cells are divided into two categories according to a WHO classification: Langerhans cell histiocytosis and Langerhans cell sarcoma. It is the degree of atypia and clinical aggressiveness that distinguishes the two subtypes. Langerhans cell sarcoma (LCS) is a neoplastic proliferation of Langerhans cells which can occur in skin, bone marrow, lymph nodes, spleen, liver, and lung. LCS can present with multiple cutaneous and systemic lesions. We present a case of Langerhans cell sarcoma with a unique morphological appearance and variable immunohistochemical profile within a single cutaneous lesion. LCS is a rare malignancy and its diagnosis is based on morphology, immunophenotypical examination, and ultrastructural analysis by electron microscopy. Our case highlights a unique morphological description of LCS wherein the pleomorphic neoplastic cells show epidermotropism and are surrounded by a variable amount of inflammatory infiltrate within a single cutaneous lesion. A single cutaneous lesion of Langerhans cell sarcoma with variable immunohistochemical profile has not been described so far.

## 1. Introduction

Langerhans cell sarcoma (LCS) is considered to be a high grade neoplasm which may present with a single cutaneous lesion or with extensive systemic involvement [1]. Typically, involvement of multiple sites is seen [2]. The tumour cells have an overtly malignant cytomorphology and it is only the immunophenotype and/or the ultrastructure which may suggest and confirm Langerhans cell lineage [3]. Primary cutaneous LCS is very rare and making an accurate histological diagnosis can be challenging [4]. Langerhans cells express CD1a, S100, and Langerin (CD207). They also show Birbeck granules on electron microscopy [5].

A number of differential diagnoses, all of which can have an overtly malignant cytomorphology, need to be considered before making a diagnosis of LCS. These include metastatic carcinoma, malignant melanoma, anaplastic large cell lymphoma, pleomorphic dermal sarcoma, atypical fibroxanthoma, and myeloid sarcoma [3]. We present a case of LCS which shows morphological and immunohistochemical variability within a single cutaneous lesion of LCS, highlighting the difficulty of making this rare diagnosis in small punch biopsies.

## 2. Case Presentation

A 91-year-old man presented with a long standing 3 cm plaque on his back which had recently become ulcerated. This lesion contained three distinct circular areas. The first area was fleshy, the second area was indurated with some telangiectasia, and the third area was ulcerated with adjacent telangiectasia and induration. There was no history of weight loss, organomegaly, lymphadenopathy, or any other systemic symptoms. Similar skin lesions were not present elsewhere.

Each of the lesions was biopsied. After a diagnosis of Langerhans cell sarcoma was made, the patient underwent a wide local excision of the biopsy site and also underwent a staging CT scan. Imaging revealed no evidence of involvement of other organ systems. A decision was made to manage the patient conservatively without further therapeutic intervention. The patient has been followed up for 24 months and there has been no evidence of recurrent disease.

The biopsies and surgical specimens were fixed in 10% buffered formalin. These were embedded in paraffin blocks. Four micron thick sections were cut and stained with haematoxylin and eosin (H and E). The antibodies in this study (CD3, CD30, CD15, CD1a, S100, CD45, CD21, CD68, CD20, ALK1, CD2, CD7, CD5, HMB-45, Melan A, and Myeloperoxidase) were obtained from DAKO and the immunohistochemistry was undertaken according to the manufacturer's instructions. Langerin immunohistochemistry was undertaken at a referral centre. A portion of the tumour was submitted for electron microscopic examination.

**Figure 1:**
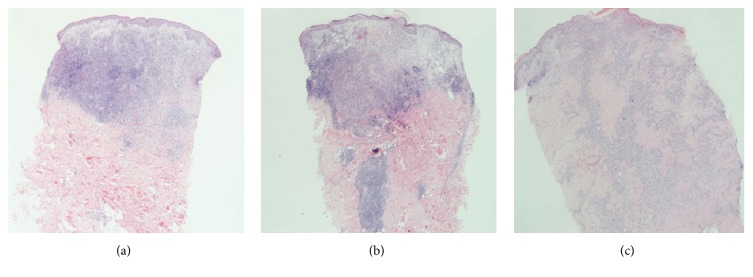
Low power H&E images demonstrating variable amount of inflammatory infiltrate around tumour cells and extending into the subcutaneous tissue in each of the three biopsies (a), (b), and (c).

**Figure 2:**
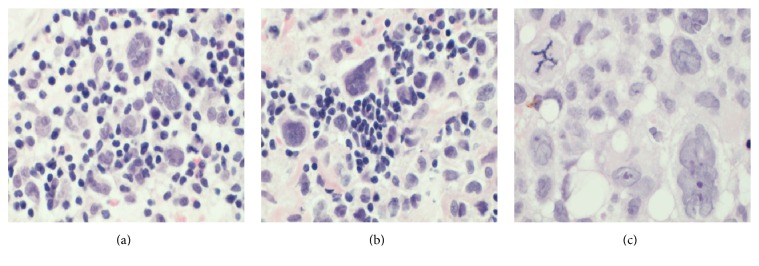
High power H&E images demonstrating variable inflammatory infiltrate surrounding neoplastic tumour cells in each of the three biopsies (a), (b), and (c).

**Figure 3:**
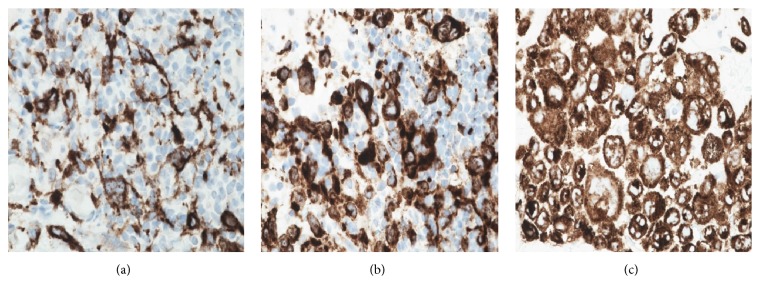
Langerin immunohistochemistry highlights tumour cells at the three biopsy sites (a), (b), and (c).

**Figure 4:**
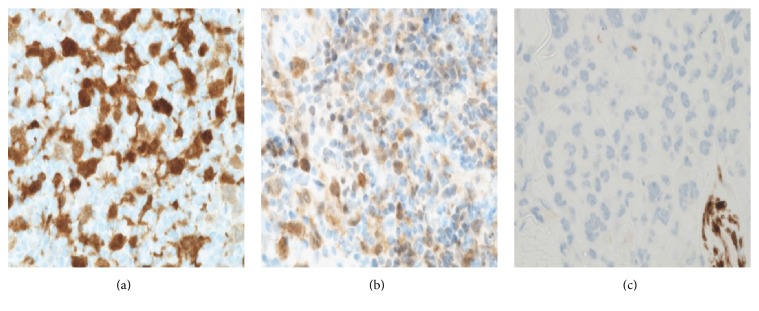
S100 immunohistochemistry positivity was variable in the three biopsies; these images compare the S100 positivity from the three different biopsy sites. (a) shows that tumour cells are S100 positive; (b) shows that occasional tumour cells are S100 positive; (c) shows that tumour cells are negative for S100. This variability within a single cutaneous lesion has not been reported within a single LCS lesion.

**Figure 5:**
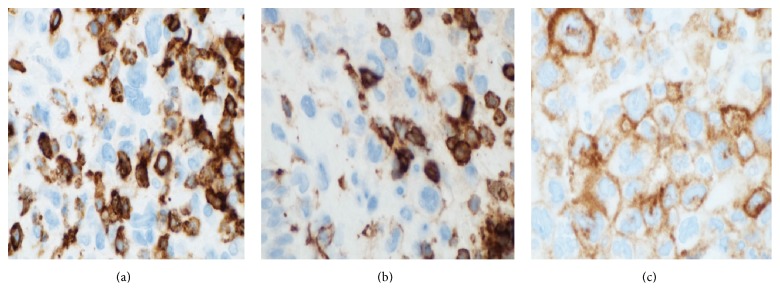
CD45 immunohistochemistry. Biopsies from the first (a) and second site (b) were positive whereas the biopsy from the third site (c) was negative.

Each biopsy showed a dermal infiltrate composed of highly atypical cells with abundant cytoplasm and bizarre nuclei, complex nuclear membrane infolding, and prominent nucleoli. The tumour cells were seen extending into the subcutaneous tissue. The neoplastic cells also showed epidermotropism. There were numerous mitoses and a variable chronic inflammatory infiltrate was admixed with the tumour cells (Figures 1 and 2). The tumour cells in all three biopsies were positive for CD1a and Langerin (Figure 3). Tumour cells from the first area biopsied were strongly positive for S100, whereas the cells from the second area biopsied showed weak positivity for S100 and from the third area they were negative with S100 (Figure 4). There was equivocal staining in the first and second biopsies with CD45 but strong positive staining in the third biopsy (Figure 5).

**Figure 6:**
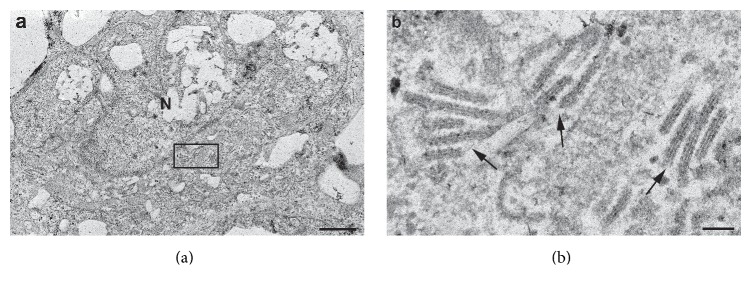
Electron microscopy demonstrates Birbeck granules. Birbeck granules are racquet or rod shaped cell organelles which are a characteristic feature of Langerhans cell. The presence of these on ultrastructural analysis confirms that the cell of origin is from the dendritic cell family.

Myeloperoxidase, CD21, CD68, CD20, CD3, CD30, CD15, Alk1, CD2, CD7, CD5, CD56, and pan melanoma cocktail (HMB45 and Melan A) were negative. The staining for Mib1 revealed heterogeneity in the proportion of tumour cells staining with this antibody within the dermal and intraepidermal components of the tumour. Focally the dermal component shows 50–80% positivity. The intradermal component cannot be accurately enumerated because of the sparsity of the tumour cells and also because an absolute distinction cannot be made from staining in epithelial cells without recourse to double labelling with CD1a and Mib1 which we are unable to do. The presence of Birbeck granules was confirmed by electron microscopy, confirming the Langerhans cell lineage of the tumour (Figure 6).

The excision specimen was a skin ellipse with underlying fat (5 cm × 4 cm × 4 cm) and contained several discrete plaques with a central bluish area. Tumour invaded to a depth of 6 mm. There was similar morphological and immunophenotypical variability in the tumour cells as seen in the biopsies. Histology showed that the surgical margins were clear by 4 mm.

## 3. Discussion

LCS is an extremely rare tumour arising from Langerhans cells; these cells are part of the dendritic cell family and their primary role is antigen capture and presentation [6]. These cells are normally found in the epidermis and also along with the mucosal lining of the oral cavity, pharynx, oesophagus, upper airways, urethra, and female reproductive tract [6]. LCS has a high degree of cytological atypia and usually a clinically aggressive course [2]; most cutaneous Langerhans cell sarcomas are described as having large atypical cells, some of which have an epithelioid appearance with pleomorphic nuclei; these are usually found in the dermis and the subcutaneous tissue [4]. In this case, morphologically the lesion contained pleomorphic bizarre cells with epidermotropism which has so far not been described in cutaneous Langerhans cell sarcoma. The tumour cells in LCS are reported to express CD1a, S100, and Langerin [7]. These tumours may arise de novo or may complicate myeloproliferative disorders and also rarely Langerhans cell histiocytosis [2]. Bohn et al. reviewed a total of 20 cases and reported that purely cutaneous presentation was seen in only three cases whilst the remaining cases showed involvement of both the skin and other organ systems [4]. Complete absence of expression of S100 has been reported in one case [4]. Three other cases showed equivocal staining [4]. Of note, there was evidence of significant intralesional heterogeneity in the immunophenotype of the tumour cells in this case. This variability seen with S100 and CD45 staining has not been reported previously in a LCS lesion.

It is of interest that there was a variable degree of chronic inflammation associated with the tumour in each of the biopsies raising the possibility of a host chronic inflammatory cell response to the neoplasm.

Although S100 negative cells have been described in previous case reports [4], a review of cases suggests that a large proportion of tumours will show S100 positivity in more than 50% of the neoplastic cells [7]. This immunohistochemical variability within a single lesion could potentially make the diagnosis of LCS more challenging. In our case, the neoplastic cells were positive with CD1a and Langerin.

Our case highlights a number of unique features of cutaneous Langerhans cell sarcoma. The presence of a longstanding plaque may hypothetically indicate the possibility of an initial in situ lesion prior to invasion into the dermis and subcutaneous tissue. This case demonstrates a unique morphology of Langerhans cell sarcoma with large atypical pleomorphic cells demonstrating epidermotropism with tumour cells surrounded by a variable amount of inflammatory infiltrate within the dermis. The immunohistochemical variability seen within this single cutaneous lesion highlights that the immunohistochemical profile of this tumour may still be incomplete and the immunohistochemical variability could potentially make this diagnosis challenging if a limited panel of antibodies is used.

## Abbreviations

LCS: Langerhans cell sarcoma
CD: Cluster of differentiation
H and E: Haematoxylin and Eosin.

## References

[1] Valentín-Nogueras S. M., Seijo-Montes R., Montalván-Mirõ E., Sánchez J. L. (2013). Langerhans cell sarcoma: A case report. *Journal of Cutaneous Pathology*.

[2] Sagransky M. J., Deng A. C., Magro C. M. (2013). Primary cutaneous Langerhans cell sarcoma: A report of four cases and review of the literature. *American Journal of Dermatopathology*.

[3] Wang C., Chen Y., Gao C., Yin J., Li H. (2012). Multifocal Langerhans cell sarcoma involving epidermis: a case report and review. *Diagnostic Pathology*.

[4] Bohn O. L., Ruiz-Argüelles G., Navarro L., Saldivar J., Sanchez-Sosa S. (2007). Cutaneous Langerhans cell sarcoma: A case report and review of the literature. *International Journal of Hematology*.

[5] Nakayama M., Takahashi K., Hori M. (2010). Langerhans cell sarcoma of the cervical lymph node: A case report and literature review. *Auris Nasus Larynx*.

[6] Chikwava K., Jaffe R. (2004). Langerin (CD207) staining in normal pediatric tissues, reactive lymph nodes, and childhood histiocytic disorders. *Pediatric and Developmental Pathology*.

[7] Pileri S. A., Grogan T. M., Harris N. L. (2002). Tumours of histiocytes and accessory dendritic cells: an immunohistochemical approach to classification from the international lymphoma study group based on 61 cases. *Histopathology*.

